# A literature-derived dataset on risk factors for dry eye disease

**DOI:** 10.1038/s41597-023-01931-8

**Published:** 2023-01-11

**Authors:** Wanju Yang, Kuiliang Yang, Yumiao Pan, Shangcao Wu, Xiangxi Chen, Lei Shen, Qingyan Zeng, Jianhua Wu, Minzhi Lv, Junyan Zhang, Yanning Yang, Yiqiao Xing

**Affiliations:** 1 Aier Eye Hospital of Wuhan University, Wuhan, Hubei 430063 China; 2 Eye Center, Renmin Hospital of Wuhan University, Wuhan, Hubei 430060 China; 3 Department of Biostatistics, Zhongshan Hospital, Fudan University, Shanghai, 200032 China; 4 Center of Evidence-Based Medicine, Fudan University, Shanghai, 200433 China; 5 Department of Clinical Study Designing, Bothwin Clinical Study Consultant, Redmond, WA 98053 USA

**Keywords:** Risk factors, Eye diseases

## Abstract

Dry eye disease (DED) is a common disease associated with disorder of tear secretion. Research on risk factors for DED, such as depression, arthritis, thyroid disease, stroke and diabetes, is important to facilitate its diagnosis and prognosis. We created a dataset on risk factors for DED (DrDED) with public access that can provide up-to-date and validated data acquired from systematically searched and screened, high-quality studies. The established DrDED contained 119 studies published between 2000 and 2022. The range of the study sample size was from 43 to 4,871,504. The study types were, as follows: cross-sectional (*n* = 92), retrospective cohort (*n* = 9), prospective cohort (*n* = 10), and case-control (*n* = 8) studies. Data from eligible studies were collected and presented for the present study, including the publication information, study characteristics, definition and prevalence of the disease, and risk factors for DED, together with the strength of association. With the publication of new relevant studies, the DrDED will be updated, and the data will be made accessible to the users.

**Table:** 

Design Type(s)	Dataset creation objective
Measurement Type(s)	Patient outcome • scientific publication • risk factors • dry eye disease
Technology Type(s)	Digital curation • documenting • meta-analysis
Factor Type(s)	Depression • arthritis • thyroid disease • stroke disease • diabetes
Sample Characteristic(s)	Homo sapiens • dry eye disease • global

## Background & Summary

Dry eye disease (DED) is an inflammatory and chronic clinical condition, and characteristic for reduced tear film stability that affects the ocular surface^1,2^. DED occurs when any of the three layers of a healthy tear film (aqueous fluid, mucus, and fatty oils) is affected, and as a result, the surface of the eyes is inadequately lubricated, and became less smooth and clear^3^.

The prevalence of DED is high among ophthalmic conditions, and varies between 14.6% and 30%^4^, depending on the diagnostic method used and population characteristics^5^. A recent systematic review revealed that one of five Asians experience DED^6^. Hence, DED has been considered a disease of multifactorial etiology, and is correlated to the presence of certain comorbidities in systemic organs^7^. DED etiology and pathology findings have indicated that patients with different DED etiologies may present with different signs and symptoms^8^. It has been reported that patients who smoke or have comorbidities experience more severe symptoms of DED, when compared to patients in a control group^9^. The potential factors that might increase the incidence of DED include primary lacrimal gland diseases, rheumatoid arthritis, ultraviolet ray exposure, improper wearing of contact lenses, older age, female, smoking, and some medicines, such as tricyclic antidepressants and antihypertensive drugs^3,6,10–12^.

The categories of risk factors for DED can be classified as modifiable and non-modifiable, and further classified into three categories: consistent, probable and inconclusive^13^. For example, a modifiable factor, alcohol drinking, was considered inconclusive, in terms of its association with DED. On the contrary, Asian ethnicity is considered an essential, non-modifiable and consistent risk factor for DED^14^. The observed increase in incidence of DED in recent years may be associated with the growing exposure to risk factors in the general population, such as increased sunlight exposure, smoking, and aging^12,15^. The examination of DED risk factors and the strength of its association with DED can provide valuable information for the prevention and treatment of DED.

The present study established a dataset on risk factors for dry eye disease (DrDED^16^), which comprised of published literatures of high-quality clinical studies that reported the DED risk factors and its associative strengths. At present, the DrDED contains the following data obtained from 119 studies: publication details, geographic regions from where the study subjects were obtained, number and demographic characteristics of the study subjects, the DED risk factors under investigation, and the magnitude of the association strength with the disease in the form of statistical measures, including odds ratios (ORs) and confidence intervals (CIs).

## Methods

### Overview and literature searches

**Fig. 1:**
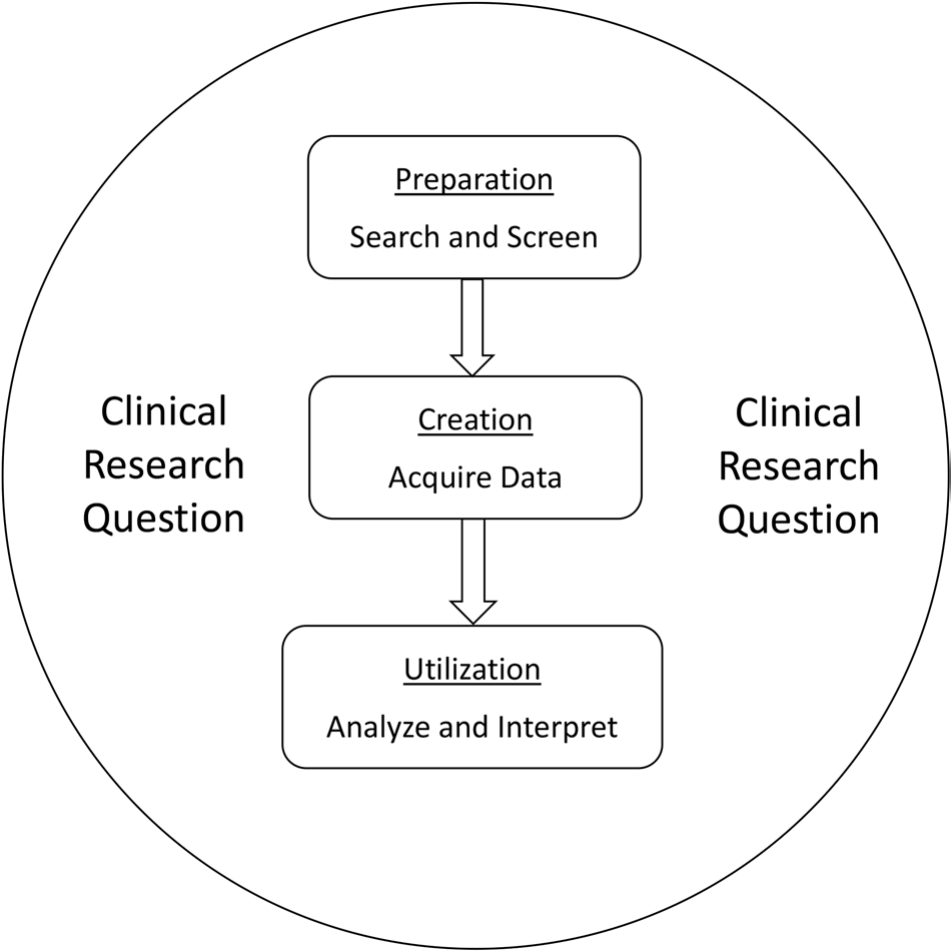
Diagram for the development of the DrDED. DrDED, dataset on risk factors for dry eye disease.

The present study was conducted based on the Preferred Reporting Items for Systematic Reviews and Meta-Analyses guidelines^10^. The clinical research question was initially formulated: what are the risk factors of DED and the strength of its association with the disease? The preparation stage for the DrDED development included a systematic literature search, the establishment of the inclusion and exclusion criteria, and the study screening through the evaluation of the eligibility of each study. Next, the DrDED was created by acquiring data correlated to the research question from eligible studies, and entering these data in a Microsoft Excel spreadsheet. After the data validation, the dataset was ready for utilization by researchers who had access to the DrDED. All stages were conducted with focus on the clinical research question (Fig. 1).

A systematic search strategy was performed using the following Medical Subject Headings terms and free-text terms: “dry eye syndrome”, “dry eye disease”, “dry eye”, “evaporative dry eye disease or syndrome”, “population at risk”, “risk factor”, “risk score”, and “health correlate” (the relationship among disease names and among risk factor-related terms, “OR”; the relationship between the disease and risk factors). The Medline (since 1946), Embase (since 1974), and Cochrane Central Register of Controlled Trials (since 1996) electronic databases were also searched from inception to September 2022. No restriction was made on the type of study. In order to ensure that no eligible study was missed, the references of previously published systematic reviews^11^ and included studies were checked.

### Inclusion and exclusion criteria

The following inclusion criteria were applied for the screening of the titles, abstracts and full-text articles: any type of primary patient research on risk factors or predictors for DED, including cross-sectional studies, case-control studies and cohort studies; DED was diagnosed according to the United States National Eye Institute/Industry Workshop criteria^12^; the data for risk factors in the DED analysis was provided; the appropriate methods for the study quality control were reported; a between-group comparability analysis was performed for the baseline data; confounding variables were adjusted in the calculation of ORs for the studied factors; complete study and participant information (author/s, year of publication, country, participant age, and gender) was available. Articles that reported duplicate data from the same study were excluded.

### Risk of bias assessment

Two independent researchers performed the risk of bias assessment for each included study using the risk of bias tool, the Newcastle-Ottawa Scale (NOS), as recommended by the Cochrane Handbook for Systematic Reviews. The NOS consisted of eight items in three domains (selection, comparability and outcomes), and the total score ranged from 0 to 9. The studies were rated, as follows: low risk of bias (high quality study), when the NOS score ranged within 7–9; high risk of bias, when the NOS score ranged within 4–6; very high risk of bias (very low quality study), when the NOS score ranged within 0–3^17^.

## Data Records

**Fig. 2:**
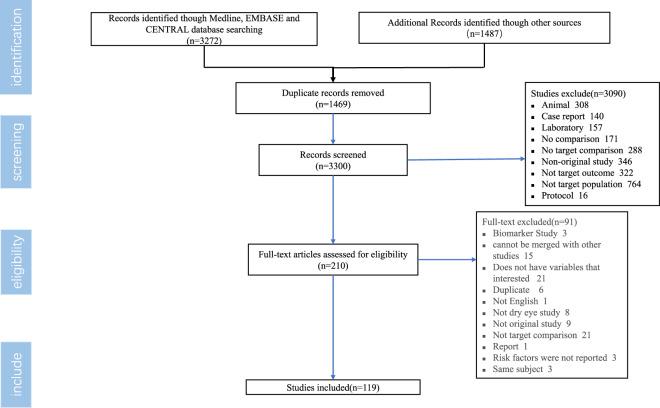
Study eligibility assessment flow diagram for the DrDED. DrDED, dataset on risk factors for dry eye disease.

As presented in Fig. 2, among the 3,300 citations found in the extensive search, 210 articles were identified after screening the titles and abstracts, and after reviewing the full texts. A total of 119 relevant articles published between 1997 and 2022 met the study inclusion criteria^16^. The types of studies were, as follows: cross-sectional (*n* = 92), case-control (*n* = 8), retrospective cohort (*n* = 9), and prospective cohort (*n* = 10) studies. The range of the study sample size was from 43 to 4,871,504. The reported risk factors and strength of association with the disease (measured by multiple logistic regression models and reported with ORs and 95% CIs) were acquired from eligible studies, and studies included in the DrDED^16^. Table 1 presents the structure of the DrDED. The data obtained from all eligible studies, according to the clinical research question, was entered in the database^16^.

**Table 1:** Structure of the DrDED.

Category	Data items
A. Study publication information	1. Study unique ID, including the subgroup labels where applicable
	2. The study record, which comprises of the last name of the first author and year of publication, and the full citation
	3. Full name of the first author
	4. Year of publication
	5. Name the journal where the study was published
B. Study characteristics	6. Type of study with the following options: cross-sectional, case-control, nest case-control, retrospective cohort, and prospective cohort studies.
	7. Risk of bias assessment
	8. Country where the study was conducted
	9. Sample population characteristics, such as population-based, hospital-based, and university-based population.
	10. Age of the study subjects
	11. Number of enrolled subjects
	12. Ethnicity
C. Definition and prevalence of the disease	13. Diagnosis standard used to define the DED in the study
	14. Prevalence of DED (point estimate and values of variability)
D. Risk factors	15. Central tendency estimate (in the form of OR) of each risk factor reported by the study*
	16. Lower and higher bounds of association between the risk factor and disease with 95% CIs for the ORs
	17. Subgroups, if any**.

Note: DrDED, dataset on risk factors for dry eye disease; ID, identifier; DED, dry eye disease; OR, odds ratio; CI, confidence interval. ^*^The data was reported as categorical variables, unless specified. ^**^When the subgroup data is available, this will be entered in the DrDED in multiple rows and the study ID will be labelled with the number and information of the subgroups.

A total of 34 risk factors were reported in the DrDED including thyroid disease, arthritis, hypertension, diabetes, stroke disease, smoking status, alcohol drinking status, depression, age (every 10 years, as a continuous variable for continuous variables or categorical variables as reported in the original studies), gender, caffeine use, antihistamines use, visual display terminal use, contact lens use, body mass index, site/geographical region, gout history, dyslipidemia, multivitamin use, pterygium, occupation or work activity environment, allergy, hormone replacement therapy, corticosteroid use, cataract or cataract surgery, glaucoma, residence at high altitude, under-correction of refractive error/refractive surgery, residence (rural or urban), benign prostatic hyperplasia, rosacea, diuretics, migraine headache, and sleep disorders.

In order to illustrate the utilizations of the DrDED, a subset of 41 representative studies published between January 2000 and April 2021, which reported one or more of the eight common risk factors for DED, was selected. In these studies, the participants were recruited from the same geographic location where the respective studies were conducted, and the baseline characteristics of participants between the case and control groups were comparable. When the OR and 95% CI values did not include the no-effect threshold of 1, a significant association with DED was indicated, and the factor was considered a risk factor of DED.

**Fig. 3:**
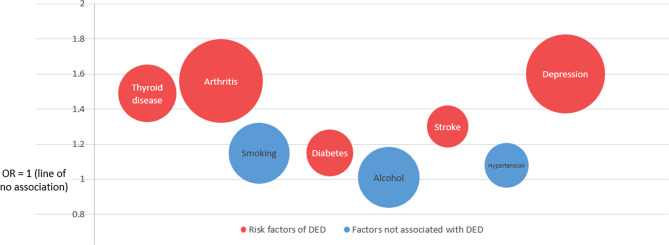
Risk factors and the association strength for DED (a subset of high-quality studies between 2000 and 2020, which reported one or more of the eight common risk factors for DED). DED, dry eye disease. The further away the bubble was from the line of no association (OR [odds ratio] = 1), the stronger the association strength became. The red bubbles represent the risk factors, and the blue bubbles represent the factors not associated with DED.

The synthesized data demonstrated that participant comorbidities (from the highest to the lowest strength of the association) of depression (OR, 1.60; 95% CI, 1.34 to 1.92), arthritis (OR, 1.56; 95% CI, 1.28 to 1.91), thyroid disease (OR, 1.49; 95% CI, 1.34 to 1.66), stroke (OR, 1.30; 95% CI, 1.21 to 1.39), and diabetes (OR, 1.15; 95% CI, 1.04 to 1.27) were risk factors of the DED; whereas hypertension (OR, 1.08; 95% CI, 0.99 to 1.18), smoking (OR, 1.14; 95% CI, 0.98, 1.33), and alcohol use (OR, 1.01; 95% CI, 0.84 to 1.20) were not associated with the DED (Table 2, Fig. 3).

**Table 2:** Overview for the subset data records in the DrDED (41 high-quality studies between 2000 and 2021 that reported one or more of the eight common risk factors for DED).

Patient factor	Study IDs	Overall assessment	Prospective cohort studies	Cross-sectional studies	Case-control studies	Retrospective cohort studies
Thyroid disease	3, 16, 29, 32, 48, 51, 58, 68, 85, 95, 97, 146	1.49-fold risk increase	1.27-fold risk increase	1.55-fold risk increase	—	No association
Arthritis	3, 10, 29, 32, 42, 48, 51, 68, 75, 81, 85, 95, 97, 103	1.56-fold risk increase	No association	1.51-fold risk increase	2.16-fold risk increase	—
Hypertension	22, 26, 32, 46, 48, 51, 57, 58, 67, 71, 75, 81, 85, 97, 103, 105, 127, 135, 147	No association	No association	No association	—	No association
Diabetes	3, 42, 51, 58, 67, 68, 71, 75, 81, 85, 92, 97, 103, 147	1.15-fold risk increase	No association	1.13-fold risk increase	1.41-fold risk increase	No association
Stroke disease	26, 75, 97	1.3-fold risk increase	—	1.3-fold risk increase	—	—
Smoking	3, 4, 18, 24, 28, 32, 36, 51, 54, 58, 60, 61, 71, 72, 81, 92, 97, 105, 147	No association	1.74-fold risk increase	No association	No association	No association
Alcohol use	24, 32, 57, 61, 81, 97	No association	—	No association	1.85-fold risk decrease (protective factor)	—
Depression	28, 32, 42, 56, 58, 75, 85, 92, 97, 98, 117, 135, 140, 146	1.6-fold risk increase	—	1.61-fold risk increase	1.45-fold risk increase	1.77-fold risk increase

The association and strength of the association between the reported risk factors and DED are listed in the table. For example: patients with thyroid disease were 1.49-fold more likely to develop DED, when compared to patients without the disease (OR, 1.49; 95% CI, 1.34–1.66); patients with diabetes were 1.15-fold more likely to develop DED (OR, 1.15; 95% CI, 1.04–1.27). DrDED, dataset on risk factors for dry eye disease; ID, identifier;--, no data; OR, odds ratio; CI, confidence interval.

## Technical Validation

The gold-standard two-step process of two independent reviewers were conducted based on the consensus between the products of the two reviewers, and this was strictly implemented throughout the entire creation process of the DrDED. Two experienced reviewers with ophthalmologic expertise backgrounds screened the titles and abstracts, screened the full-texts, and independently acquired the data from eligible studies (Fig. 1). Each datapoint in the documents of the two reviewers was manually compared and validated using the statistical software, Review Manager (RevMan, Version 5.4, the Cochrane Collaboration, 2020). The accuracy of the extracted sample sizes, and the OR and 95% CI values were presented by generating a forest plot in both numeric and graphic forms. If any discrepancy was identified in the data and outliers reported by the two reviewers, the full text was carefully reviewed, and the data was corrected, accordingly. Any disagreements on the study eligibility was resolved through team discussion. Furthermore, an external methodologist conducted the final data validation by checking the study flow, logic and accuracy of all data.

## Usage Notes

The DrDED can serve its users from two aspects. First, the data can be used to investigate the risk factors of DED through the subgroup meta-analysis of different age groups (younger *vs*. older), gender, countries/continents, sample population sources (population-based *vs*. specific institution-based), study sample size (large *vs*. small), the diagnosis criteria of DED, and other subgroup categories, when reported by primary studies. The user can use the data for further statistical analysis, such as Egger’s test, in order to determine whether publication bias is present^14^. Second, the DrDED serves as a library and a research planning source for users. For clinical researchers who wants to conduct their own epidemiological study to explore the risk factors of DED in a population, they can use the information in the dataset to assist in the study design, in terms of the study subject’s inclusion criteria, determination of sample sizes, questionnaire design, and data to be collected. When needed, they can also refer to the details in the data analysis methods and background knowledge by reviewing the full texts of high-quality studies that were systematically searched and screened for the clinical research question.

The DrDED is a living dataset, because the information is updated through data search and acquisition on a yearly basis, and every time a well-known study is published. The procedures described in the present study will be applied, including the data validation, when adding data obtained from new publications to the DrDED, with focus on the risk factors for DED, thereby keeping the dataset up-to-date. The DrDED is publicly available on the journal platform in its electronic version. Its future up-to-date versions can be obtained by contacting the corresponding authors of the present study *via* E-mail.

## Supplementary information

Data Citation 1 DrDED

Data Citation 2 Study Selection

## Data Availability

Not applicable.
